# *WallProtDB*, a database resource for plant cell wall proteomics

**DOI:** 10.1186/s13007-015-0045-y

**Published:** 2015-01-16

**Authors:** Hélène San Clemente, Elisabeth Jamet

**Affiliations:** Université de Toulouse; UPS; UMR 5546, Laboratoire de Recherche en Sciences Végétales, BP 42617, F-31326 Castanet-Tolosan, France; CNRS; UMR 5546, BP 42617, F-31326 Castanet-Tolosan, France

**Keywords:** Cell wall, Mass spectrometry, Plant, *ProtAnnDB*, Proteomics

## Abstract

**Background:**

During the last fifteen years, cell wall proteomics has become a major research field with the publication of more than 50 articles describing plant cell wall proteomes. The *WallProtDB* database has been designed as a tool to facilitate the inventory, the interpretation of cell wall proteomics data and the comparisons between cell wall proteomes.

**Results:**

*WallProtDB* (http://www.polebio.lrsv.ups-tlse.fr/WallProtDB/) presently contains 2170 proteins and ESTs identified experimentally in 36 cell wall proteomics studies performed on 11 different plant species. Two criteria have to be met for entering *WallProtDB*. First one is related to the identification of proteins. Only proteins identified in plant with available genomic or ESTs data are considered to ensure unambiguous identification. Second criterion is related to the difficulty to obtain clean cell wall fractions. Indeed, since cell walls constitute an open compartment difficult to isolate, numerous proteins predicted to be intracellular and/or having functions inside the cell have been identified in cell wall extracts. Then, except proteins predicted to be plasma membrane proteins, only proteins having a predicted signal peptide and no known intracellular retention signal are included in the database. In addition, *WallProtDB* contains information about the strategies used to obtain cell wall protein extracts and to identify proteins by mass spectrometry and bioinformatics. Mass spectrometry data are included when available. All the proteins of *WallProtDB* are linked to *ProtAnnDB*, another database, which contains structural and functional bioinformatics annotations of proteins as well as links to other databases (Aramemnon, CAZy, Planet, Phytozome). A list of references in the cell wall proteomics field is also provided.

**Conclusions:**

*WallProtDB* aims at becoming a cell wall proteome reference database. It can be updated at any time on request and provide a support for sharing cell wall proteomics data and literature references with researchers interested in plant cell wall biology.

**Electronic supplementary material:**

The online version of this article (doi:10.1186/s13007-015-0045-y) contains supplementary material, which is available to authorized users.

## Background

The plant cell wall is an external matrix containing polysaccharides and proteins. The interest in plant cell wall proteomes has been increasing during the last years with the discovery that plant cell walls are dynamic compartments constantly modified during development and in response to environmental cues [1,2]. The physiology of plant cell walls is strongly linked to its enzyme and structural protein content. The full description of the proteins present in various cell walls at precise stages of development or in response to biotic and abiotic stresses is now a main goal for many laboratories [3-5]. Besides, the search for procedures efficiently deconstructing cell walls to produce bioethanol has renewed the interest in cell wall physiology and especially in proteins playing roles in the remodeling of cell wall polysaccharides which are the major constituents of biomass [6-9].

Recent progresses in mass spectrometry (MS) technologies have led to the identification of cell wall proteins (CWPs) allowing the description of many cell wall proteomes. The next challenge is to gain biological messages out of these data. The first problem is the validation of the proteins identified as *bona fide* CWPs. This point is critical in plant cell wall proteomics. Indeed, it is difficult (i) to extract proteins by non-destructive methods avoiding the leakage of plasma membranes and the release of intracellular proteins and (ii) to purify cells walls because they form an open compartment which is not delimited by membranes [3,10]. Two kinds of methods have been employed: non-destructive methods consist in the analysis of extracellular fluids collected by vacuum infiltration of different types of solutions or of culture medium; destructive methods comprise several steps starting with the grinding of plant material followed by the purification of cell walls and the extraction of proteins with salt solutions [3]. The type of identified proteins and the ratio between identified proteins predicted to be secreted and identified leaderless proteins depends on the type of method used and on the type of plant material [10,11]. The issue of the non-canonical CWPs, *i.e.* proteins having no predicted signal peptide, has been a matter of debate since the first cell wall proteomics studies [12-14]. The second problem is the quality of functional annotations of proteins in databases. They are often not sufficiently reliable to allow an appropriate biological interpretation of proteomics data because they are mostly based on sequence comparisons [15,16]. The third problem occurs with plants for which sequence data are not available. In this case, the proteins cannot be unambiguously identified. This is a major problem in plants since most cell wall proteins belong to multigene families [17]. All these difficulties make the comparison between different cell wall proteomes a challenging task.

In order to answer such questions, *WallProtDB* (http://www.polebio.lrsv.ups-tlse.fr/WallProtDB/) was built in 2008 as a tool (i) to collect cell wall proteomics data, (ii) to facilitate their biological interpretation, and (iii) to allow comparisons between cell wall proteomes of different plant species. A new version of *WallProtDB* has been recently launched with new tools allowing the comparison between cell wall proteomes from different organs of the same plants or from different plants. *WallProtDB* contains experimental published data which are manually curated and is restricted to plants for which sequence data, genomic or ESTs, are available. Protein accession numbers are linked to another database, *ProtAnnDB* (http://www.polebio.lrsv.ups-tlse.fr/ProtAnnDB/), which provides bioinformatics predictions of sub-cellular localization and functional domains of diverse plant proteins using programs available online [15].

## Construction and content

### Construction and updating

**Figure 1:**
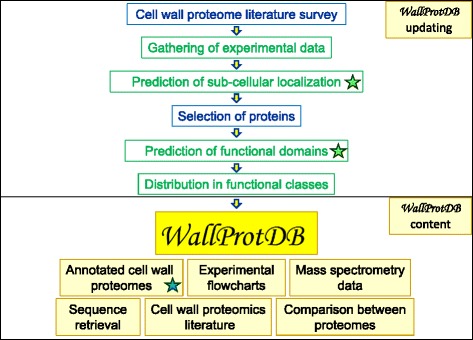
**The** ***WallProtDB*** **flowchart (upper part) and content (lower part).** *ProtAnnDB* is used at two different steps of the annotation procedure: prediction of sub-cellular localization and of functional domains (indicated by green stars). The accession number of identified protein is linked to *ProtAnnDB* (blue star).

The construction and the updating of *WallProtDB* are supported by the following steps performed to ensure a reliable database (Figure 1):

1. Literature survey of plant cell wall proteomics papers. Selection of papers describing cell wall proteomes of plants with available sequenced genomes. Gathering of experimental data.
2. Bioinformatic prediction of sub-cellular localization of proteins. This annotation is done using *ProtAnnDB* which is regularly enriched with new proteins. Depending on the plant of interest, protein sequences are from different databases (Table 1).
3. Selection of proteins having predicted signal peptide, but no intracellular retention signal such as an ER canonical retention signal (IPR011679, http://www.ebi.ac.uk/interpro/entry/IPR011679; PS00014, http://prosite.expasy.org/PS00014) and no more than one trans-membrane domain as predicted by TMHMM (http://www.cbs.dtu.dk/services/TMHMM-2.0/).
4. Bioinformatics prediction of functional domains. This annotation is done using *ProtAnnDB* (see below).
5. Definition of a dictionary for the functional annotation of proteins, based on Pfam (http://pfam.xfam.org) [18] or InterPro (http://www.ebi.ac.uk/interpro/) [19] domain repertoires. This step ensures that the same annotation is used for all the proteins sharing the same predicted functional domains.
6. Classification of proteins into 8 functional classes on the basis of the presence of predicted functional domains [10]: proteins acting on cell wall polysaccharides, oxido-reductases, proteases, proteins related to lipid metabolism, proteins with interaction domains (with proteins or polysaccharides), proteins possibly involved in signaling, structural proteins, proteins with yet unknown function. All the other proteins are included in a ninth class named “miscellaneous proteins” (Additional file 1).
7. Design of a flowchart form allowing the description of most of the possible strategies usable to isolate CWPs and to identify them by MS and bioinformatics [3]. Customize the form for each set of experimental data (for an example, see Analysis of the cell wall proteome of *Brachypodium distachyon* young leaves: http://www.polebio.lrsv.ups-tlse.fr/WallProtDB_data/biblio/biblio26.html).
8. Addition of MS data when available using either the X!Tandem software [20] or links to excel sheets found as supplementary data in articles of interest. When the data are in the X!Tandem format, it is possible to visualize the sequenced peptides on the protein sequence and their MS/MS fragmentation data.
9. Search for homologous proteins in closely related genomes when only ESTs are available. The identification of homologous genes allows completing the bioinformatics prediction of signal peptide and/or of functional domains when EST sequences are not full-length. This is the case for *Saccharum officinarum* and *Brassica oleracea* for which homologous genes have been searched for in *Sorghum bicolor* and *Arabidopsis thaliana* respectively.
10. Addition of cell wall proteomics literature. Direct links to articles or to their abstract in PubMed-NCBI (http://www.ncbi.nlm.nih.gov/pubmed) are available.

**Table 1:** **Proteomes included in** ***WallProtDB***

**Plant species**	**Common name**	**Sequence databases**	**Proteome size** ^**a**^
*Arabidopsis thaliana*	Thale cress	TAIR10 http://www.ncbi.nlm.nih.gov/	495
*Brachypodium distachyon*	False brome	http://www.brachypodium.org/database	314
*Brassica oleracea*	cabbage	COMPBIO http://compbio.dfci.harvard.edu/index.html	162 (ESTs)
*Gossypium hirsutum*	cotton	COTTONGEN http://www.cottongen.org/search/search_by_gene	116
*Linum usitatissimum*	flax	Genolin flax unigenes https://urgi.versailles.inra.fr/Species/Flax/Download-sequences	106
		Phytozome http://www.phytozome.net/	
*Medicago sativa*	Alfalfa	JCVI: *Medicago truncatula* Genome Project http://medicago.jcvi.org/medicago/index.php	199
*Oryza sativa*	Rice	RAPD http://rapdb.dna.affrc.go.jp	270
*Populus spp*	Poplar	DOE Joint Genome Institute http://genome.jgi-psf.org/Poptr1_1/Poptr1_1.home.html	142
		Phytozome http://www.phytozome.net/	
*Saccharum officinarum*	Sugarcane	SUCEST http://www.sucest-fun.org/index.php/overview/sucest-fun-project	69 (ESTs)
*Solanum lycopersicum*	Tomato	sol genomics network http://solgenomics.net/	161
*Solanum tuberosum*	Potato	COMPBIO http://compbio.dfci.harvard.edu/index.html	136

^a^ESTs mean that protein identification was done using translated ESTs because of the lack of genomic data. Note that the distribution of proteins into functional classes is indicated in Additional file 1 for the cell wall proteome of all the plant species listed in this table.

Data are stored in a mySQL database. *WallProtDB* is queried through a web interface constructed in the PHP code (http://www.php.net/).

## Bioinformatics annotation of proteins using *ProtAnnDB*

*ProtAnnDB* is an annotation tool used for (i) selecting proteins to be included in *WallProtDB* and (ii) providing annotation of selected proteins. *ProtAnnDB* collects the results of bioinformatics predictions of sub-cellular localization and functional domains using available programs [17]. The following programs or databases have been used for prediction of sub-cellular localization: SignalP (http://www.cbs.dtu.dk/services/SignalP/) [21], TargetP (http://www.cbs.dtu.dk/services/TargetP/) [22], Predotar (http://urgi.versailles.inra.fr/predotar/predotar.html) [23], Aramemnon (http://aramemnon.botanik.uni-koeln.de/) [24], TMHMM (http://www.cbs.dtu.dk/services/TMHMM-2.0/), GPIsom (http://gpi.unibe.ch/) [25] and PredGPI (http://gpcr.biocomp.unibo.it/predgpi/pred.htm) [26]. The databases used for the prediction of functional domains are Pfam [18], InterPro [19] and PROSITE (http://prosite.expasy.org/) [27]. *ProtAnnDB* also offers links to other databases providing genomic or gene regulation data such as Phytozome which collects genomic data (http://www.phytozome.net) and PlaNet which provides co-expression networks (http://aranet.mpimp-golm.mpg.de/) [28]. PlaNet has been chosen because it gives information on all the *A. thaliana* genes as well as on other plant species. *ProtAnnDB* has also links to Aramemnon which presently contains membrane protein data for nine plant species (http://aramemnon.botanik.uni-koeln.de/index.ep) and to two databases which collect expert annotation on cell wall protein families: (i) the PeroxiBase which is dedicated to peroxidases (http://peroxibase.toulouse.inra.fr/) [29] and (ii) CAZy which provides annotation of carbohydrate active enzymes (http://www.cazy.org/, http://csbl.bmb.uga.edu/dbCAN/) [30,31].

## Tools for browsing *WallProtDB*

They are three ways to query *WallProtDB*: “Detailed search”, “Summarized search” or “Blast search”.

**Figure 2:**
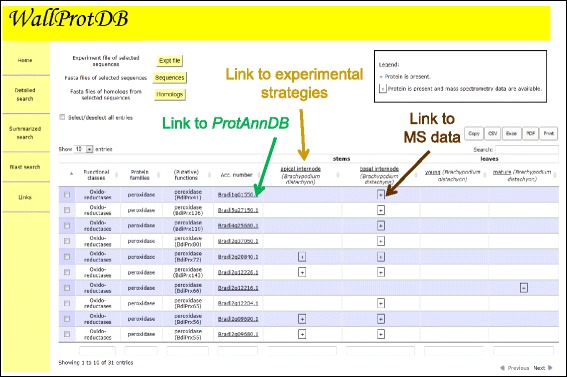
**An example of results of a Detailed search:** ***B. distachyon*** **as a plant; peroxidases as a protein family; leaves and stems as plant organs.** Note that only a part of the result sheet is shown.

**Figure 3:**
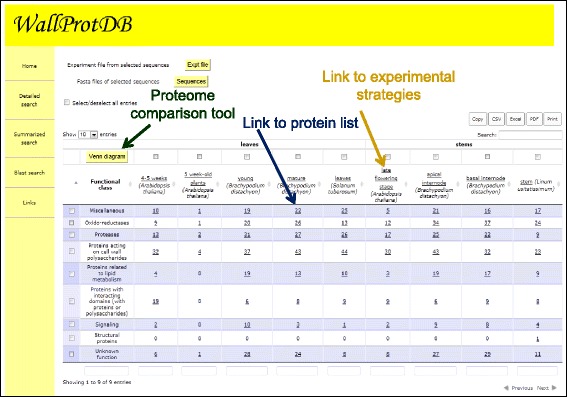
**An example of results of a Summarized search performed using the following criteria: group by functional class;** ***A. thaliana*** **,** ***B. distachyon*** **,** ***L. usitatissimum*** **and** ***S. tuberosum*** **as plants; leaves and stems as plant organs.**

**Figure 4:**
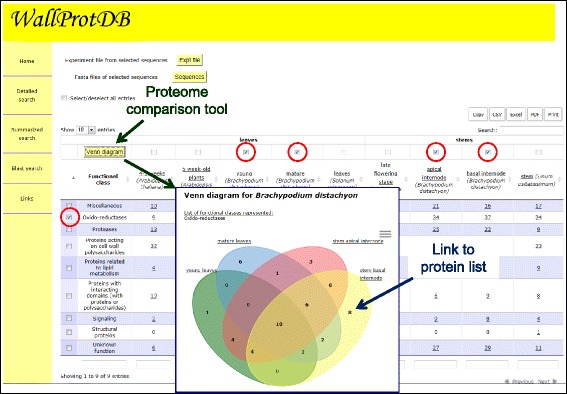
**An example of comparison between different proteomes within a plant species using the Summarized search.** The same criteria as in Figure 3 were used to obtain a table of results. Then, the Venn diagram was obtained after selection of *B. distachyon* as a plant, oxido-reductases as a functional class, and the four following experiments: young leaves (green), mature leaves (blue), apical internodes (pink), basal internodes (yellow).

1. The “Detailed search” interface offers several criteria: (1) protein accession number; (2) plant species; (3) plant material; (4) protein functional class; (5) protein family; (6) keyword. These criteria can be combined to refine comparisons. The result of the query is a customizable table that can be exported in different formats such as a tab delimited text, an excel sheet or a pdf file (Figure 2). Alternatively, they can be directly printed. Hyperlinks lead to *ProtAnnDB* bioinformatics annotation, experimental flowcharts and MS data (Figure 2). Protein sequences can be retrieved in FASTA format.
2. The “Summarized search” interface provides tools for overall proteome comparisons. The result of the query is a table in which the numbers of proteins in each (i) protein functional class, (ii) protein family or (iii) protein (putative) function are indicated (Figure 3). As mentioned above, different formats are available for export of query results. It is also possible to draw a Venn diagram to visualize proteome comparisons within a plant species (Figure 4). All the figures are clickable, thus enabling retrieval of lists of the corresponding proteins.
3. The “Blast search” [32] permits finding sequences homologous to a given nucleic or protein sequence in *WallProtDB*. A list of hits is proposed together with the possibility to visualize sequence comparisons and to collect the protein sequences in the FASTA format. It allows clustering newly identified CWPs with proteins present in the database. Then, it is easier to link the presence of some protein clusters to different physiological conditions and/or to cell wall types.

## Utility and discussion

At present, *WallProtDB* contains 2170 proteins and expressed sequence tags (ESTs) identified in 36 cell wall proteomics studies performed on 11 different plant species (8 dicots and 3 monocots) (Table 1, Additional file 1). It also offers tools for comparisons between proteomes. *WallProtDB* is regularly updated with newly published experimental data which are manually curated to obtain a homogeneous annotation (prediction of sub-cellular localization and functional domains of proteins). Only proteins having a signal peptide to address proteins to the secretion pathway and no known intracellular retention signal are included in the database. Proteins predicted to be plasma membrane proteins have been introduced in the database such as cellulose synthase, callose synthase or receptor kinases. They have been identified through peptides located in their extracellular domain. They are not true CWPs, but since they are involved in cell wall biogenesis or in signal transduction, they might be of interest for people working in the plant biology field. In addition, *WallProtDB* contains information about the protocols used to obtain cell wall protein extracts and about the strategies to identify proteins by MS and bioinformatics, as well as MS data when available. Furthermore, *WallProtDB* provides a list of references in the cell wall proteomics field which is regularly updated and comprises experimental articles as well as reviews.

*WallProtDB* complements other databases such as SUBcellular location database for Arabidopsis proteins (SUBA3, http://suba.plantenergy.uwa.edu.au/) [33], The Plant Proteome Database (PPDB, http://ppdb.tc.cornell.edu/) [34] and the cellwallgenomics database (http://cellwall.genomics.purdue.edu/intro/index.html). In each of these databases, the gathering of information is done in a different way. In SUBA3, only *A. thaliana* proteins are listed and all the proteins identified in published proteomes are included. This can be misleading because proteins known to be intracellular can be found in proteomes called “cell wall proteomes”. For example, the At5g38410 *A. thaliana* small subunit of RUBISCO is mentioned as an extracellular or a plasma membrane protein although it is a well described chloroplastic protein. However, since all the proteins identified in cell wall proteomes are listed, SUBA3 is useful to get access to leaderless proteins identified in cell wall proteomes. PPDB is devoted to *A. thaliana*, *Oryza sativa* and *Zea mays*. It contains experimental MS data on proteins identified in different organs or sub-cellular compartments including the cell wall. Finally, the cellwallgenomics database provides repertoires of genes involved in cell wall biogenesis in *A. thaliana*, *O. sativa*, *S. bicolor* and *Z. mays* including intracellular proteins such as glycosyl transferases involved in the biosynthesis of cell wall polysaccharides. It also gives information on some mutants and on techniques useful to study cell wall biology, but no cell wall proteomics data.

## Conclusions

To date, *WallProtDB* describes the content of cell wall proteomes and proposes tools for their analysis. It contains proteins with a high probability of being *bona fide* CWPs with regard to our present knowledge of the secretion pathway and of cell wall physiology. However, in the future, it could also include proteins with no predicted signal peptides, but experimentally proven to be located in cell walls by alternative methods such as localization of proteins tagged with fluorescent proteins or immunolabeling [3]. So far, there are only a few examples of such proteins in plants. The symplastic mannitol deshydrogenase has been shown to be secreted upon pathogen infection, and the secretion can occur in the presence of brefeldin A. However, the mechanism of secretion has not been described [35]. The exocyst-positive organelle (EXPO) could mediate the exocytosis from cytosol to cell wall of learderless proteins such as SAMS2 (S-adenosylmethionine synthetase 2) [36]. Since all the proteins included in *WallProtDB* are annotated in the same way, it allows fine comparisons between cell wall proteomes of different species and of various plant materials. In addition, it allows clustering proteins on a sequence homology basis. New proteomes can be introduced in *WallProtDB* on request, providing EST or genomic sequences of the plant of interest are available. The distribution of proteins into functional classes will certainly evolve when the functions of the proteins are experimentally determined. New functional classes can be easily created. Finally, the possibility to introduce wall proteomes of plant pathogens or symbionts will be considered since they share common protein families with plant cell wall proteomes. Altogether, *WallProtDB* aims at becoming a cell wall proteome reference database.

## Availability and requirements

WallProtDB is freely available at the following address: http://www.polebio.lrsv.ups-tlse.fr/WallProtDB/. It is compatible with all major web browsers.

## Additional file

Additional file 1
**The** ***WallProtDB*** **content.**
