# Supplementary material for: WallProtDB, a database resource for plant cell wall proteomics
Source: Plant Methods. 2015 Jan 16;11:2. doi: 10.1186/s13007-015-0045-y (PMC4302427; doi:10.1186/s13007-015-0045-y)
Supplement: Additional file 1 — The WallProtDB content. [file 13007_2015_45_MOESM1_ESM.pdf]

Additional file 1

Altogether, *WallProtDB* contains 2170 proteins which have been classified into functional classes according to Jamet *et al.* (2008).

Functional classes	Proteins acting on cell wall polysaccharides	Oxido-reductases	Proteases	Proteins related to lipid metabolism	Proteins with interacting domains (with proteins or polysaccharides)	Proteins possibly involved in signaling	Structural proteins	Proteins with yet unknown function	Miscellaneous proteins	Total number of proteins
**Dicots**										
Arabidopsis thaliana	128	71	58	31	52	33	8	61	53	495
Brassica oleracea	48	39	28	8	8	9	0	9	13	162
Gossypium hirsutum	39	19	9	9	3	13	0	8	16	116
Linum usitatissimum	23	24	9	9	8	4	1	11	17	106
Medicago sativa	43	34	29	13	30	14	2	13	21	199
Populus spp	48	33	20	13	1	7	0	13	7	142
Solanum lycopersicum	41	17	26	19	22	5	1	10	20	161
Solanum tuberosum	44	13	26	10	9	1	0	8	25	136
**Monocots**										
Brachypodium distachyon	73	52	45	28	17	14	0	50	35	314
Oryza sativa	83	36	32	26	22	7	8	26	30	270
Saccharum officinarum	8	21	6	3	8	1	0	8	14	69
**Total number of proteins**	578	359	288	169	180	108	20	217	251	2170
